# MYRiAD: a multi-array room acoustic database

**DOI:** 10.1186/s13636-023-00284-9

**Published:** 2023-04-26

**Authors:** Thomas Dietzen, Randall Ali, Maja Taseska, Toon van Waterschoot

**Affiliations:** 1 Department of Electrical Engineering (ESAT), STADIUS Center for Dynamical Systems, Signal Processing and Data Analytics, KU Leuven, Leuven, Belgium; 2 Microsoft, Munich, Germany

**Keywords:** Room acoustic database, Room impulse response, Cocktail party noise, Microphone array, Loudspeaker array, Acoustic signal processing

## Abstract

In the development of acoustic signal processing algorithms, their evaluation in various acoustic environments is of utmost importance. In order to advance evaluation in realistic and reproducible scenarios, several high-quality acoustic databases have been developed over the years. In this paper, we present another complementary database of acoustic recordings, referred to as the Multi-arraY Room Acoustic Database (MYRiAD). The MYRiAD database is unique in its diversity of microphone configurations suiting a wide range of enhancement and reproduction applications (such as assistive hearing, teleconferencing, or sound zoning), the acoustics of the two recording spaces, and the variety of contained signals including 1214 room impulse responses (RIRs), reproduced speech, music, and stationary noise, as well as recordings of live cocktail parties held in both rooms. The microphone configurations comprise a dummy head (DH) with in-ear omnidirectional microphones, two behind-the-ear (BTE) pieces equipped with 2 omnidirectional microphones each, 5 external omnidirectional microphones (XMs), and two concentric circular microphone arrays (CMAs) consisting of 12 omnidirectional microphones in total. The two recording spaces, namely the SONORA Audio Laboratory (SAL) and the Alamire Interactive Laboratory (AIL), have reverberation times of 2.1 s and 0.5 s, respectively. Audio signals were reproduced using 10 movable loudspeakers in the SAL and a built-in array of 24 loudspeakers in the AIL. MATLAB and Python scripts are included for accessing the signals as well as microphone and loudspeaker coordinates. The database is publicly available (https://zenodo.org/record/7389996).

## Introduction

Acoustic signal processing using multiple microphones has received significant attention due to its fundamental role in a number of applications such as assistive hearing with hearing aids or cochlear implants, teleconferencing, hands-free telephony, voice-controlled devices, spatial audio reproduction, and sound-zoning, just to name a few. Some of the specific tasks which can be accomplished with acoustic signal processing include speech enhancement and speech dereverberation [1–8], room parameter estimation [9], acoustic echo and feedback cancelation [10, 11], source localization [2, 5, 12], audio source separation [7, 8], sound field control [13, 14], and automatic speech recognition [15], all of which are pertinent to the aforementioned applications. One of the core phases in the development of acoustic signal processing algorithms is that of the evaluation phase, where the performance of a newly developed algorithm is compared to that of existing algorithms in various acoustic environments which are relevant for the application at hand. This is clearly challenging because the laboratory conditions under which the algorithm is evaluated rarely match the real-world conditions where the algorithm must perform. Additionally, recorded audio signals with the target microphone configurations and specified acoustic scenarios may be unavailable, resulting in the use of simulated data for evaluation. Although simulated data can be useful in the evaluation of initial proof of concept ideas, it does not necessarily provide accurate indication whether the algorithm will perform well in real-world conditions. In an effort to overcome these challenges and to encourage the use of more realistic data, several high-quality acoustic databases containing room impulse responses (RIRs) [6, 9, 17–27], speech [6, 9, 10, 15, 20, 22, 23], music [20], and babble or cocktail party noise [22, 28, 29] have been developed over the years, which have played an important role in building confidence in the real-world performance of various acoustic signal processing algorithms.

In this paper, we present another complementary database of acoustic recordings from multiple microphones in various acoustic scenarios, referred to as the Multi-arraY Room Acoustic Database (MYRiAD). In comparison to the existing databases, the MYRiAD database is unique in its diversity of the employed microphone configurations suiting a wide range of applications, the acoustics of the recording spaces, and the variety of signals contained in the database, which includes RIRs, recordings of reproduced speech, music, and stationary noise, as well as recordings of live cocktail parties.

The database consists specifically of two different microphone configurations used across two different rooms. The first microphone configuration consists of a dummy head (DH) with in-ear omnidirectional microphones, two behind-the-ear (BTE) pieces mounted on the DH, each equipped with 2 omnidirectional microphones,^1^ as well as 5 external omnidirectional microphones (XMs) located at various distances and angles from the DH.^2^ This microphone configuration will be referred to as M1. The second microphone configuration consists of two concentric circular microphone arrays (CMAs) with in total 12 omnidirectional microphones,^3^ which will be referred to as M2. The two different rooms where audio recordings were made are as follows: (i) the SONORA Audio Laboratory [35] located at the Department of Electrical Engineering (ESAT-STADIUS), KU Leuven, Belgium, which we will refer to as the SAL, and (ii) the Alamire Interactive Laboratory [35] located at the Park Abbey in Heverlee, Belgium, referred to as the AIL. The main acoustical difference between these two rooms is that the SAL is significantly more reverberant than the AIL, with reverberation times of 2.1 s and 0.5 s, respectively. In the SAL, the microphone configuration M1 was used in one position, and in the AIL, a combination of microphone configurations M1 and M2 was used in two positions. In terms of sound generation, 10 different movable loudspeakers were used as artificial sound sources in the SAL, while the AIL has been equipped with an array of 24 loudspeakers.

**Fig. 1:**
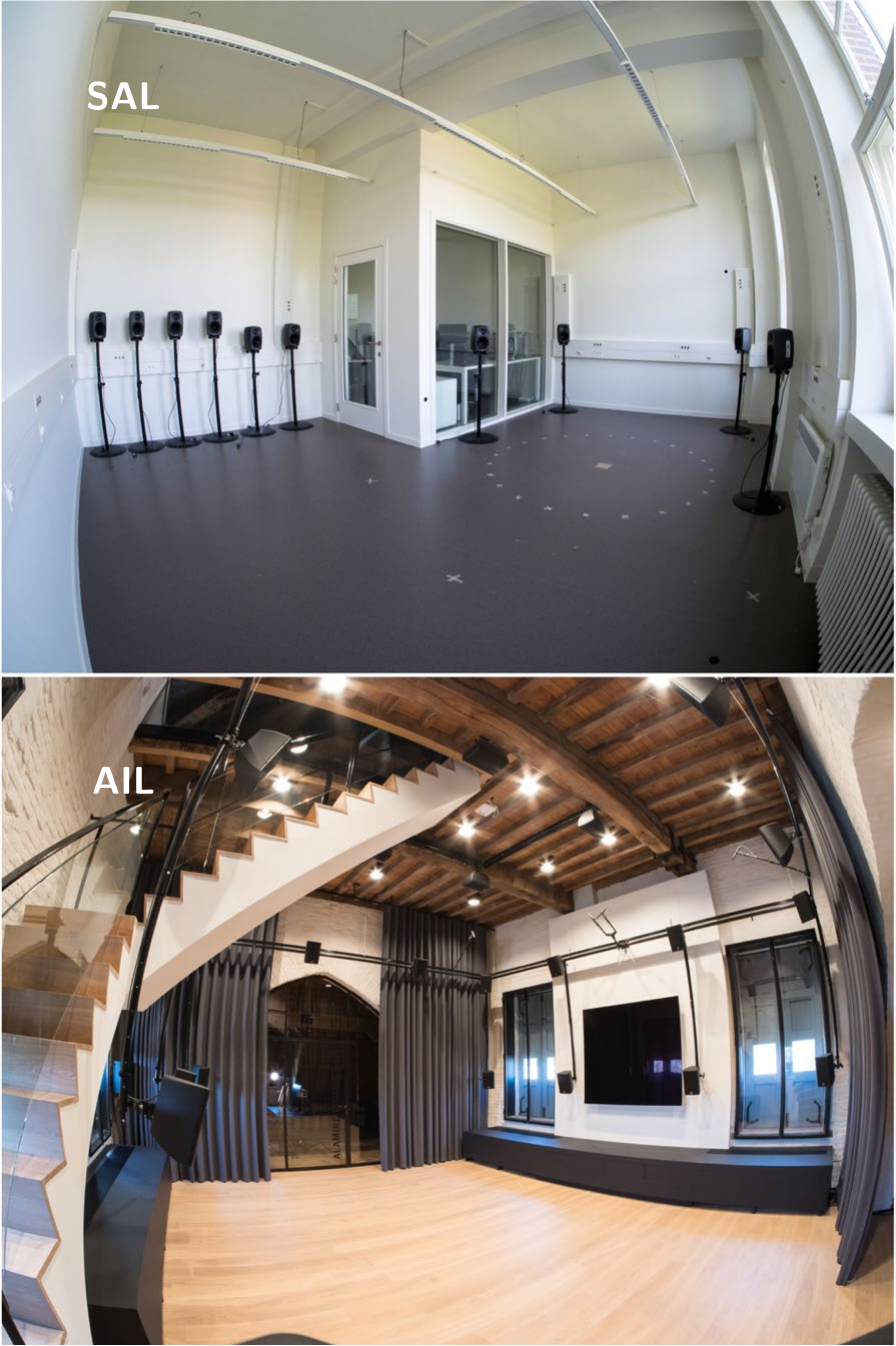
Fisheye view of the SAL and the AIL

**Table 1:** Equipment used for creating the database

Type			Product	Room/Mic. Config.
**Hardware**	**Reproduction**	**Loudspeakers**	Genelec 8030 CP	SAL
			Martin Audio CDD6	AIL
		**DA-converters**	RME M-32 DA	SAL
			Powersoft OTTOCANALI 4K4 DSP+D	AIL
	**Acquisition**	**Microphones**	Neumann KU-100 DH	M1
			BTE left/right-ear pieces from Cochlear	M1
			AKG CK97-O	M1
			AKG CK32	M1 & M2
			DPA 4060	M2
		**AD-converters/pre-amplfiers**	RME Micstasy	SAL & AIL
			Proprietary pre-amp. for BTE microphones	SAL & AIL
	**Digital interface**		RME Digiface USB audio interface	SAL
			Ferrofish Verto 64	AIL
			Apple iMac	SAL & AIL
**Software**	**Reproduction/acquisition**		Logic Pro X	SAL
			Adobe Audition	AIL
	**Post-processing**		MATLAB	
			Python	

The following audio signals were played back through the speakers and recorded by the microphones: exponential sine sweeps used to compute RIRs [36] between source and microphone positions, resulting in 110 RIRs for the SAL and 1104 RIRs for the AIL, as well as three male speeches [37], three female speeches [37], a drum beat [38], a piano piece [39], and speech-shaped stationary noise. Additionally, in both rooms, several participants were invited to re-create a live cocktail party scenario. The resulting noise from the different cocktail parties held at each of the spaces was recorded for both microphone configurations.

In total, the MYRiAD database contains 76 h of audio data sampled at 44.1 kHz in 24 bit, which results in 36.2 GB. All computed RIRs and recorded signals are available in the database and can be downloaded [43]. MATLAB and Python scripts are included in the database for accessing the signals and corresponding microphone and loudspeaker coordinates.

The remaining sections of this paper provide a detailed overview of the database and are organized as follows. In Section 2, an overview of the two different rooms, the SAL and the AIL, is presented. In Section 3, a detailed description is given of the equipment used. In Section 4, the microphone and loudspeaker configurations within the two rooms are discussed. In Section 5, an overview is given of the recorded signals, details of the cocktail party, and the computed RIRs. In Section 6, practical instructions for using the database are provided, along with a description of relevant MATLAB and Python scripts, and some examples from the database are illustrated. In Section 7, the database is briefly summarized.

**Fig. 2:**
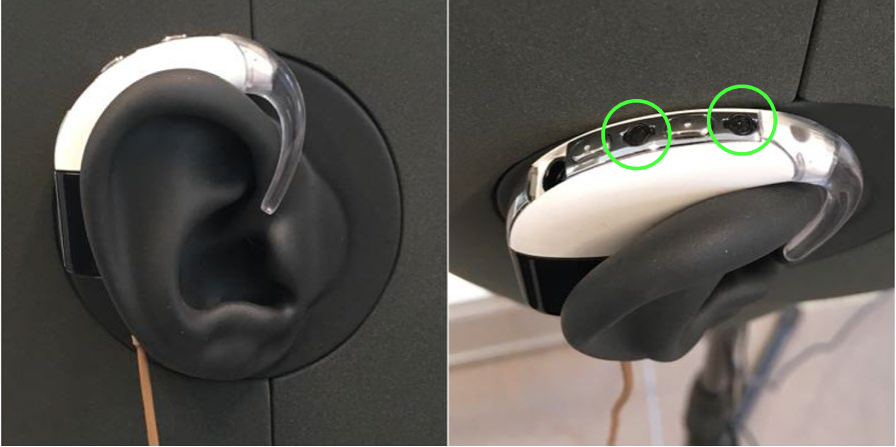
Dummy BTE pieces used for creating the database. Each BTE piece consists of two omnidirectional microphones as indicated by the circles

**Fig. 3:**
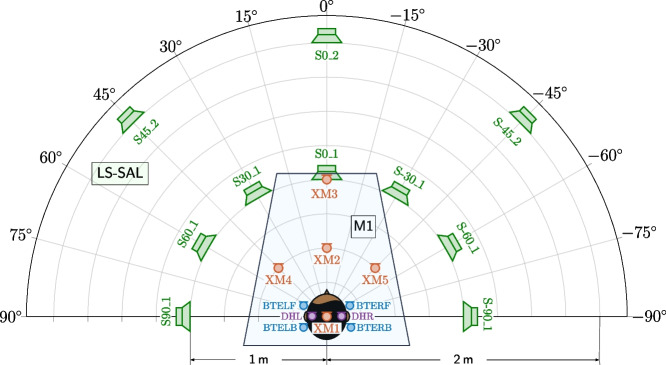
Plan view of the M1 microphone configuration and the LS-SAL loudspeaker configuration. A description of the microphone and loudspeaker labels is given in Table 2. The radial grid spacing of the polar plot is 0.25 m. The DH is placed at a height of approximately 1.3 m ear level from the floor and all XMs are placed at a height approximately 1 m from the floor. The trapezoidal shape is used to represent the M1 microphone configuration in the floor plans of Fig. 6. For extracting the coordinates of the microphone and loudspeaker positions, the MATLAB or Python scripts discussed in Section 6.2 should be used

## Room description

In this section, we provide a brief overview on the characteristics of the two recording rooms. The SAL is described in Section 2.1 and the AIL is described in Section 2.2.

### SONORA Audio Laboratory (SAL)

The SAL [35] is located at the Department of Electrical Engineering (ESAT-STADIUS), KU Leuven, Heverlee, Belgium. Figure 1 shows a fisheye view and Fig. 6 shows a floor plan of the L-shaped SAL with approximate dimensions. The height of the room is 3.75 m, yielding a volume of approximately 102 m$^{3}$. The walls and ceiling are made of plasterboard covering mineral wool, while the floor is made of concrete covered with vinyl. Two windows, each of 4 m$^{2}$, are located on one side of the room. Adjacent to the recording room, separated by glass of area 6.5 m$^{2}$, is the control room, where all the acquisition equipment and a computer are located. From the RIRs measured in the SAL, we estimated the reverberation time $\mathrm {T_{20}}$ to be 2.1 s as described in Section 6.4. Details on the audio hardware used in the SAL are given in Section 3, while the microphone and loudspeaker configuration and placement are described in Section 4.1.1, Section 4.2.1, and Section 4.3.

### Alamire Interactive Laboratory (AIL)

The AIL [35] is located in a historic gate building, the Saint Norbert’s gate of the Park Abbey in Heverlee, Belgium. Figure 1 shows a fisheye view and Fig. 6 shows a floor plan of the room. Apart from a staircase leading to a floor above, the room is approximately shoe-box shaped with 6.4 m width, 6.9 m depth, and 4.7 m height, yielding a volume of approximately 208 m$^{3}$. The floor and ceiling are made of wood. The room is closed by thin line plastered brick walls with two windows each to the front and the back of about 3.3 m$^{2}$ each, and wide passages to adjacent rooms, with one of them closed by a glass door. These passages were closed off with curtains during recording, except for a part of the cocktail party noise, cf. Section 5.3. The housing of the staircase is plastered, the stairs are wooden, and the railing is made of glass. From the RIRs measured in the AIL, the reverberation time $\mathrm {T_{20}}$ is estimated to be 0.5 s, cf. Section 6.4. The AIL is equipped with a permanent, fixed array of 24 loudspeakers for spatial audio reproduction as shown in Fig. 1. Further details on the audio hardware used in the AIL are given in Section 3, while the microphone and loudspeaker configuration and placement are described in Section 4.1.1, Section 4.1.2, Section 4.2.2, and Section 4.3.

## Recording equipment

A list of the recording and processing equipment used to create the database is shown in Table 1. In regard to the microphones, the DH contains 2 in-ear omnidirectional microphones (one for each ear) and the two BTE pieces (one for each ear) are each equipped with 2 omnidirectional microphones. The BTE pieces and their proprietary pre-amplifier were provided by Cochlear Ltd. and shown in Fig. 2. The specific loudspeaker and microphone configurations used for the various recordings in the database will be outlined in Section 4, and naming conventions of files will be defined in Section 6.

The recording chains were built as follows. As the digital audio workstations for sending and acquiring the signals, Logic Pro X and Adobe Audition on an iMac were used in the SAL and the AIL, respectively. In the SAL, the signals were sent from Logic Pro X via USB to the RME Digiface, then to the RME M-32 DA using the ADAT protocol, and finally to the respective Genelec 8030 CP loudspeakers. In the AIL, the signals were sent from Adobe Audition via the DANTE protocol to the Powersoft OTTOCANALI 4K4 DSP+D and finally to the Martin Audio CDD6 loudspeakers. In both rooms, all microphone signals were sent to an RME Micstasy (except for the BTE microphone signals which were firstly routed to the proprietary pre-amplifier) and converted to ADAT. In the SAL, the ADAT signals were sent to the RME Digiface and finally recorded on Logic Pro X, whereas in the AIL, the ADAT signals were sent to the Ferrofish Verto 64 and via DANTE to Adobe Audition. The various types of recorded signals are outlined in Section 5. For post-processing (such as RIR computation, cf. Section 5), MATLAB and Python were used.

## Microphone and loudspeaker configurations

This section describes the microphone configurations in Section 4.1, the loudspeaker configurations in Section 4.2, and the placement of these configurations within the SAL and AIL in Section 4.3. The exact coordinates of the loudspeaker and microphone positions within the SAL and AIL from the various configurations can be loaded from the database, but the details of this procedure will be elaborated upon in Section 6.

### Microphone configurations

#### M1

The first microphone configuration, M1, consists of the in-ear microphones from the DH, the microphones from the BTE pieces, three AKG CK97-O microphones, and two AKG CK32 microphones. As the AKG CK97-O and AKG CK32 microphones are not mounted on the DH, they are considered to be “external” in relation to the DH and hence will be referred to as external microphones (XMs). This M1 configuration was used in both the SAL and the AIL, cf. Section 4.3. Figure 3 depicts the plan view of the measurement configuration of the loudspeakers and microphones used for the audio recordings made in the SAL. For now, however, we will focus only on the trapezoidal shape enclosing the microphones, which is a depiction of the M1 configuration. A description of the corresponding microphone labels is given in Table 2.

For this M1 configuration, the DH is placed at a height of approximately 1.3 m ear level from the floor. Each of the BTE pieces is mounted on the DH as shown in Fig. 2. The XMs are placed^4^ within a radius of 1 m from the DH as shown in Fig. 3. XM1, XM2, and XM3 are AKG CK97-O microphones, while XM4 and XM5 are AKG CK32 microphones. The XMs are all positioned at 1 m above the floor.

**Table 2:** Microphone and loudspeaker labels

	Mic. Type/Room	Label	Description
**Microphones**	**Dummy head**	DHL	Left ear
		DHR	Right ear
	**BTE pieces**	BTELF	Left ear, front
		BTELB	Left ear, back
		BTERF	Right ear, front
		BTERB	Right ear, back
	**External microphone**	XM[i]	With index [i] as depicted in Fig. 3
			[i] $\in$ {1, 2, ..., 5}
	**Circular microphone array**	CMA[r]_[a]	At [r] cm radius and an angle of [a]$^\circ$as depicted in Fig. 4
			[r] $\in$ {10, 20}
			[a] $\in$ {−135, −90, ..., 180}
**Loudspeakers**	**SAL**	S[a]_[d]	At angle of [a]$^\circ$and in [d] m distance as depicted in Fig. 3
			[a] $\in$ {−90, −60, −45, −30, 0, 30, 45, 60, 90}
			[d] $\in$ {1, 2}
	**AIL**	S[l][i]	At height level [l] with index [i] as depicted in Fig. 5
			[l] $\in$ {L, U, T} (indicating lower, upper, and top level)
			[i] $\in$ {1, 2, ..., 12}

**Fig. 4:**
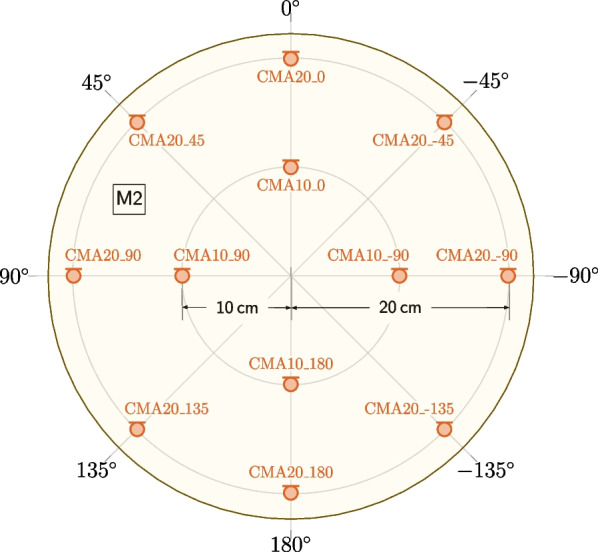
Plan view of the M2 microphone configuration. A description of the microphone labels is given in Table 2. The radial grid spacing of the polar plot is 0.1 m. DPA 4060 microphones are used for the inner circular microphone array and AKG CK32 microphones are used for the outer circular microphone array. The circle drawn around the microphones represents the M2 microphone configuration in the floor plans in Fig. 6. For extracting more precise coordinates of the microphone and loudspeaker positions, the MATLAB or Python scripts discussed in Section 6.2 should be used

**Fig. 5:**
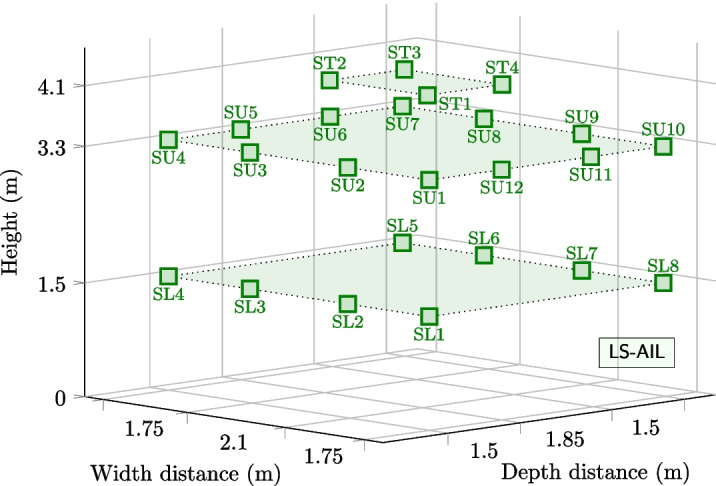
View of the LS-AIL loudspeaker array in the AIL. A description of the loudspeaker labels is given in Table 2. The speakers are organized in three different height levels of about 1.5 m (lower level), 3.3 m (upper level), and 4.1 m (top level) above the floor. The axes limits coincide with the boundaries of the approximately shoe-boxed shaped room, cf. Section 2.2. On the horizontal axes, the approximate distance between neighboring speakers is indicated. The given dimensions are of indicative nature and not exact; for extracting the coordinates of the microphone and loudspeaker positions, the MATLAB or Python scripts discussed in Section 6.2 should be used

#### M2

**Fig. 6:**
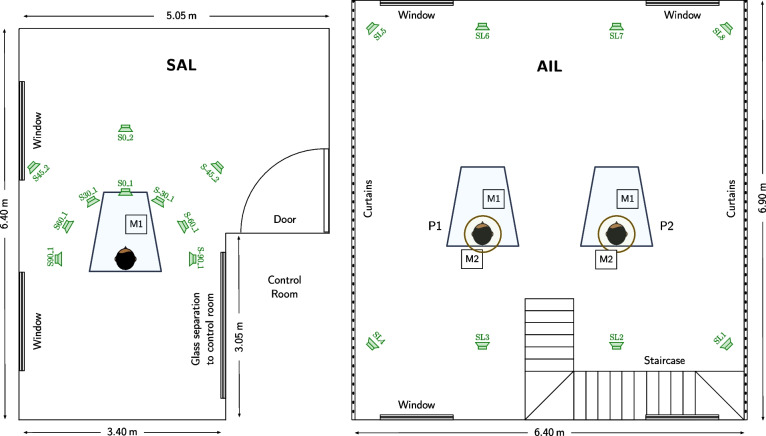
Microphone and loudspeaker configuration placement. (Left) Placement of the M1 microphone configuration and the LS-SAL loudspeaker configuration within the SAL. (Right) Placement of the M1 and M2 microphone configurations in P1 and P2 as well as the lower level of the LS-AIL loudspeaker configuration within the AIL. Details of the M1 and M2 microphone configurations and the LS-SAL and LS-AIL loudspeaker configuration can be seen in Figs. 3, 4, and 5. For extracting the coordinates of the microphone and loudspeaker positions, the MATLAB or Python scripts discussed in Section 6.2 should be used

The second microphone configuration, M2, consists of two concentric circular microphone arrays (CMAs) composed of 4 DPA 4060 and 8 AKG CK32 microphones. Figure 4 shows a plan view of the M2 configuration, and a description of the microphone labels is given in Table 2. The inner circular microphone array has a radius of 10 cm and consists of 4 equidistantly placed DPA 4060 microphones. The outer circular microphone array has a radius of 20 cm and consists of 8 equidistantly placed AKG CK32 microphones. The microphones are all placed at a height of 1 m above the floor using a holder made of laser-cut acrylic glass, centered around the stand of the DH of the M1 configuration. This M2 configuration was used at two different positions within the AIL, always in combination with M1 as depicted in Fig. 6. It should be noted that since M2 was used in combination with M1, it is also possible to define arrays that contain microphones of both configurations, such as a linear array composed of CMA20_180, CMA10_180, XM1, CMA10_0, CMA20_0, XM2, and XM3.

### Loudspeaker configurations

#### LS-SAL

The loudspeaker configuration LS-SAL as the name suggests is used in the SAL only. It is defined relative to the M1 microphone configuration, and consists of 10 loudspeakers. The loudspeakers are positioned at various spatial locations at a height such that the center of each of the woofers is approximately 1.3 m above the floor. Figure 3 is a plan view of this LS-SAL loudspeaker configuration along with the M1 microphone configuration. A description of the loudspeaker labels is also provided in Table 2. During recordings, the loudspeaker S0_1 was removed before recording the signals for the loudspeaker S0_2 so that there was a direct line of sight from the latter to the DH.

#### LS-AIL

The loudspeaker configuration LS-AIL is a 24-loud- speaker array, permanently installed in the AIL, cf. Fig. 1, which is typically used for spatial sound reproduction. Figure 5 shows the geometry of the loudspeaker array. The loudspeakers are labeled as described in Fig. 5 and Table 2. The width and depth of the array are approximately 5.6 m and 4.85 m, and the loudspeakers are arranged in three groups of different height levels, referred to as lower, upper, and top level. The lower level consists of 8 speakers located around the room along the walls at about 1.5 m height, the upper level containing 12 speakers is located above at about 3.3 m height, and the top level containing 4 speakers is located more centrally at about 4.1 m height. Note that for the sake of simplicity, the presented locations are only approximate. Using measurements of the distances between the speakers and a set of four reference points on the floor with known coordinates, the exact coordinates of the loudspeakers have been estimated based on the theory on Euclidean distance matrices [40]. All microphone and loudspeaker coordinates can be loaded from the database as discussed in Section 6.2.

### Microphone and loudspeaker configuration placement

Figure 6 illustrates the placement of the M1 microphone configuration as well as the LS-SAL loudspeaker configuration within the SAL at a recording position near the corner of the L-shaped room.

**Fig. 7:**
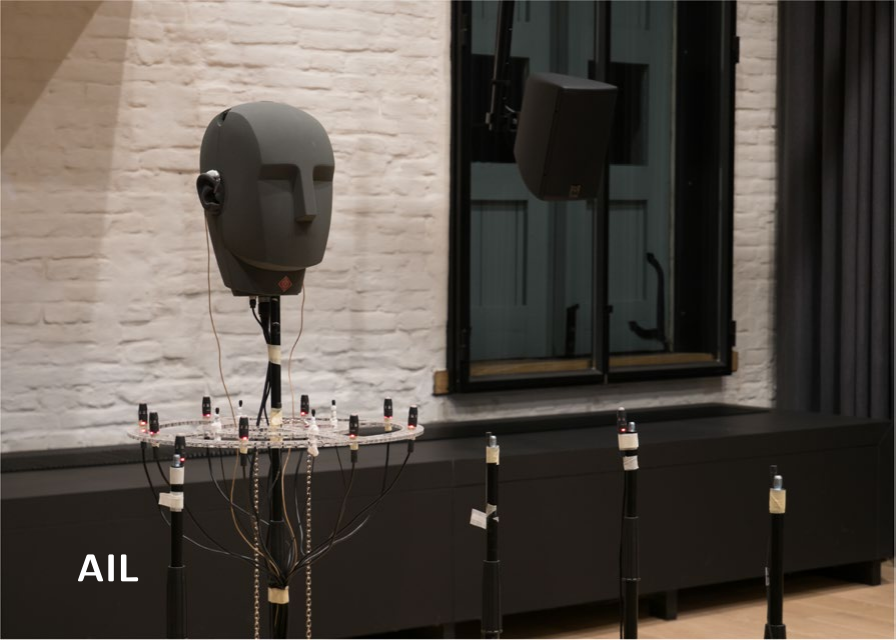
A combination of the microphone configurations M1 and M2 as used at the AIL

Figure 6 shows a floor plan of the setups M1 and M2 within the AIL, together with the lower speakers of the LS-AIL loudspeaker array. As can be seen, there are two recording positions in the AIL, referred to as P1 and P2, with the DH facing the speakers SU6 and SU7, located roughly below ST2 and ST1 (not shown in the figure), respectively. In both recording positions, both microphone configurations M1 and M2 are used, with the stand of the DH of M1 being the center of the circular microphone arrays of M2. Figure 7 shows a combination of M1 and M2 as used in position P2.

The coordinates of all speakers and microphones in both rooms can be loaded from the database using MATLAB or Python, cf. Section 6.2.

**Table 3:** Signals recorded and computed in the database

Signal	Type	Quantity	Duration (s)	Source	Acquisition	Speakers$^1$	Label
Male speaker	Speech	3	30–37	[37]	Playback + record	L$_\text {sub}$	M[i], [i] $\in$ {1, 2, 3}
Female speaker	Speech	3	30–37	[37]	Playback + record	L$_\text {sub}$	F[i], [i] $\in$ {1, 2, 3}
Stationary noise	Noise	1	35	Generated	Playback + record	L$_\text {sub}$	SN
Cocktail party	Noise	6	600	Party guests	Party + record	None	CP[i], [i] $\in$ {1, 2, ..., 6}
Drums	Music	1	41	[38]	Playback + record	L$_\text {sub}$	DR
Piano	Music	1	35	[39]	Playback + record	L$_\text {sub}$	PI
Sine sweep$^2$	Meas.	2	15	Generated	Playback + record	All	
RIR	RIR	1	2–3	Sine sweeps	Computed [36]	All	RIR

$^1$The subset L$_\text {sub}$ includes all speakers in SAL and SL1 to SL8 in the AIL, cf. Fig. 5 and Table 2.

$^2$The raw sine sweeps are not included in the database and hence do not have a label

**Fig. 8:**
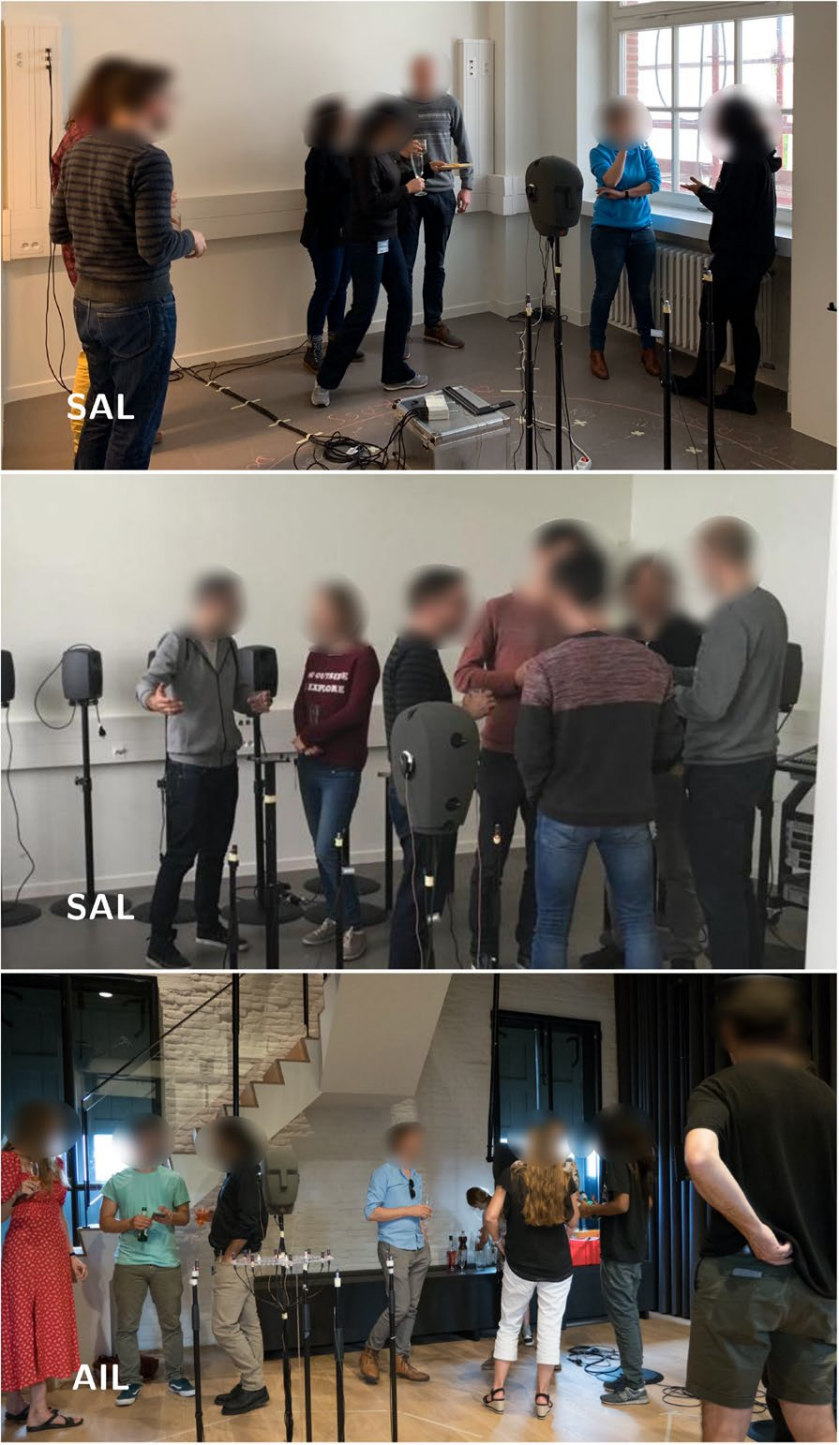
Cocktail party recordings at the SAL and the AIL

## Recorded signals

The MYRiAD database contains 76 h of audio data and has a size of 36.2 GB. All microphone signals in the database are provided at a sampling frequency of 44.1 kHz with a 24 bit resolution. Their gains are set such that the recording level across the different microphone models is approximately the same around 1 kHz in diffuse noise. For the sake of consistency, recordings were done simultaneously^5^ for all microphones in the SAL as well as in each of the two recording positions P1 and P2 in the AIL. A summary of the signals recorded and computed, along with the quantity of each (i.e., the number of different instances of that type of signal), their duration, their source, their acquisition method (i.e., how the signals were generated), the employed loudspeakers, and a signal label is provided in Table 3. In the remainder of this section, we discuss in more detail the RIR measurements in Section 5.1, the recorded speech, noise, and music signals in Section 5.2, and the recorded cocktail party in Section 5.3.

### Room impulse responses

The database includes in total 110 RIRs from the SAL and 1104 RIRs from the AIL. To obtain the RIRs, two exponential sine sweep signals were played and recorded for each loudspeaker-microphone combination. In the AIL, the sides of the room were closed off with curtains during the recording. From these sine sweeps, the RIRs were computed by cross-correlation^6^ according to the procedure detailed in [36]. From each pair of recorded sine sweeps, one of them was selected for RIR estimation by visual inspection of the spectrograms (more specifically, spectrograms containing any type of non-stationary noise were discarded). In order to obtain as clean as possible RIRs, some of the recorded sine sweeps were post-processed as to suppress low-level (stationary) harmonic noise components produced by the recording equipment. In this post-processing procedure, frequency bins containing harmonic noise components were identified during silence by comparing their magnitude to the median magnitude of neighboring frequency bins. If the difference was above the threshold of 4 dB, a Wiener filter [1] was applied in that frequency bin. The recorded signals were further post-processed to remove the input-output delay caused by the recording hardware.

### Speech, noise, music

Speech, stationary noise, and music signals were played through the loudspeakers indicated in Table 3 and recorded by all microphones. Three male and three female speech segments were chosen randomly from the Centre for Speech Technology Research (CSTR) Voice Cloning Toolkit (VCTK) corpus [37]. The stationary noise source signal has a speech-shaped spectrum and was generated in MATLAB based on speech spectra from the VCTK corpus. The drum piece was taken from the studio recording sessions in [38]. The piano piece is track 60 (Schubert) from the European Broadcast Union Sound Quality Assessment Material Recordings for Subjective Tests (EBU SQAM) [39]. In the AIL, the sides of the room were closed off with curtains during recording. These signals were acquired for all loudspeakers in the SAL, but only for the lower loudspeaker level in the AIL, that is SL1 to SL8 (in contrast to the RIRs, which were computed for all possible loudspeaker-microphone combinations, cf. Section 5.1). The recorded signals were post-processed to remove the input-output delay caused by the recording hardware. For the signals recorded in the SAL, a slow phase drift was observed between the recorded data and simulated data obtained from convolving the estimated RIR with the source signal, cf. Section 6.3. This phase drift can be associated to hardware limitations in the recording setup and has been compensated for by time-shifting some of the recorded signals^7^ such as to minimize the error between the recorded and the convolved data. For the signals recorded in the AIL, no phase drift was observed. Both the source signals and the recorded signals are included in the database.

### Cocktail party

In addition to the aforementioned signals, a cocktail party scenario was re-created and recorded in both the SAL and the AIL. All participants gave informed consent. They were instructed to stay outside of a 1 m circumference around the DH in both rooms and periodically move around in a random manner engaging in conversation. Snacks and beverages in glasses were also served to the participants during the recordings. For the SAL cocktail party, at any given time, there were at least 15 people present in the room, whereas for the AIL cocktail party, there were at least 10 and at most 14 people present. In the SAL, the microphone configuration M1 located as shown in Fig. 6 was used (the loudspeakers were removed from the room). In the AIL, the microphone configurations M1 and M2 located in position P2 as shown in Fig. 6 were used. The curtains on the sides of the room in the AIL were closed during the recordings of CP1, CP2, and CP3 and open during CP4, CP5, and CP6. Photos from the cocktail parties in the SAL and AIL are shown in Fig. 8.

**Table 4:** File path structure of the database

	**Root**		**Signal type**^**1**^		
**Source signal path**	/audio/	SRC/	[s].wav		
	**Root**	**Room**	**Speaker**^**2**^ **or CP**	**Config. placement**^**3**^	**Microphone**^**2**^ **and signal type**^**1**^
**Microphone signal path**	/audio/	SAL/	S[a]_[d]/		[m]_[s].wav
			CP/		
		AIL/	S[l][i]/	P1/	
				P2/	
			CP/	P2/	
	**Root**	**Room**			
**Coordinate file path**	coord/	SAL.csv			
		AIL.csv			
	**Root**	**Language**	**Script or function**^**4**^		
**Code file path**	/tools/	MATLAB/	[f].m		
		Python/	[f].py		

^1^The signal label [s] takes the forms as defined in Table 3

^2^The speaker labels S[a]_[d] and S[l][i] and the microphone label [m] take the forms as defined in Table 2

^3^P1 and P2 refer to the microphone configuration placements at the AIL as shown in Fig. 6

^4^The script or function names [f] take the forms as defined in Table 5

**Table 5:** Scripts facilitating the use of the database

Script or function name	Description (detailed help can be found in the header)
load_audio_data	Example script loading audio recordings and calling load_coordinates()
load_coordinates()	Function loading and optionally plotting microphone and loudspeaker coordinates

**Fig. 9:**
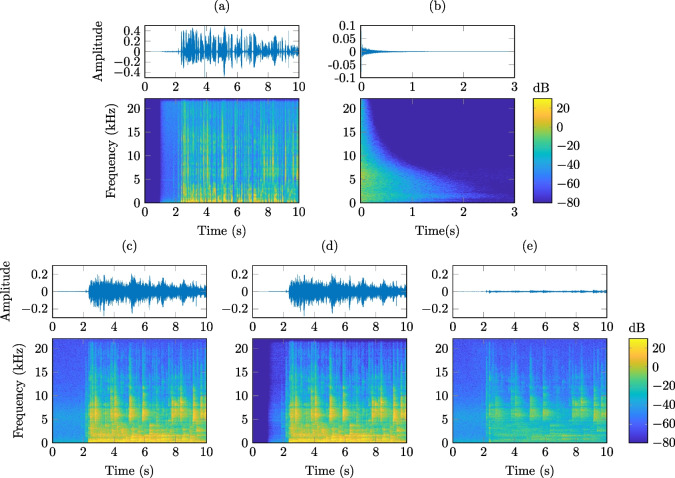
Waveform and corresponding spectrogram of signals related to the SAL recordings. **a** First 10 seconds of the source signal corresponding to a female speaker, F1 (cf. Table 3), **b** computed RIR from the loudspeaker S0_1 to microphone BTELF (cf. Fig. 3), **c** recorded microphone BTELF signal after the signal from **a** was played through the loudspeaker S0_1, **d** simulated signal from the convolution of **a** and **b**, **e** error between signals **c** and **d**

**Fig. 10:**
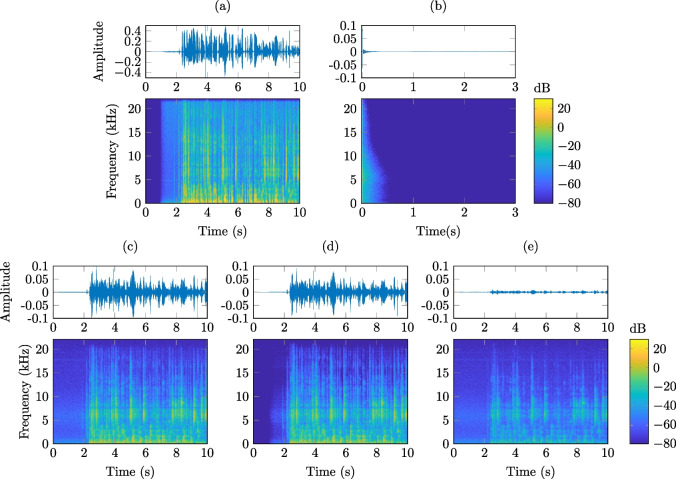
Waveform and corresponding spectrogram of signals related to the AIL recordings. **a** First 10 seconds of the source signal corresponding to a female speaker, F1 (cf. Table 3), **b** computed RIR from the loudspeaker SL5_1 to microphone BTELF (cf. Fig. 3), **c** recorded microphone BTELF signal after the signal from **a** was played through the loudspeaker SL5_1, **d** simulated signal from the convolution of **a** and **b**, **e** error between signals **c** and **d**

**Fig. 11:**
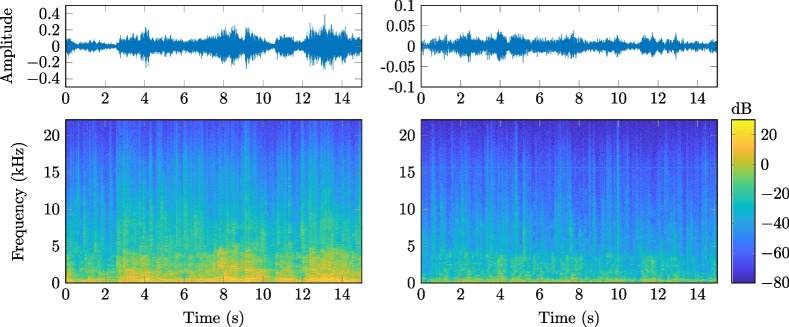
Waveform and corresponding spectrogram for a 15 s sample of the cocktail party noise. (Left) Signal CP2 for XM2 in the SAL. (Right) Signal CP5 for XM2 in the AIL

**Fig. 12:**
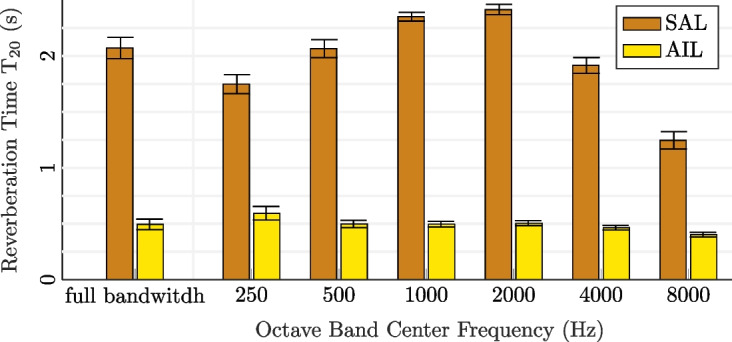
Reverberation time $\mathrm {T_{20}}$ for the two rooms SAL and AIL at full bandwidth and in different octave bands. The error bars indicate the standard deviation of the estimate across all possible loudspeaker-microphone combinations

## Using the database

In this section, we elaborate on the file path structure of the database in Section 6.1 as well as the code provided for loading audio signals and retrieving loudspeaker and microphone coordinates in Section 6.2, and present some examples of audio signals in Section 6.3 and reverberation time estimates in Section 6.4.

### File path structure

Table 4 provides an overview of the directory tree for the database. Audio files are located in the root directory /audio/, with loudspeaker source signals in the subfolder SRC/ and recorded microphone signals in the subfolders SAL/ and AIL/. The recorded microphone signals are further organized by loudspeaker (except for cocktail party recordings) and microphone configuration placement (in the AIL). The file names encode both the microphone and signal type. Note that not all folders contain all possible combinations of microphones and signals. For instance, the folder /audio/SAL/CP/ contains only files of signal type CP$^*$, and the folders in /audio/AIL/SU$^*$/ and /audio/AIL/ST$^*$/ only contain files of signal type RIR, cf. Section 5.2.

The folder /coord/ contains files with coordinates of all speakers and microphones in both the SAL and the AIL, and the folder /tools/ contain MATLAB and Python scripts for accessing audio data and coordinates, cf. Section 6.2.

### Creating microphone signals and retrieving coordinates

The database comes with MATLAB and Python scripts intended to facilitate retrieving loudspeaker and microphone coordinates and generating signals, as listed in Table 5.

The script load_audio_data is an example script demonstrating how a .wav-file can be loaded given a list of loudspeaker, microphone, and signal labels provided by the user. This script also calls the function load_coordinates(), which reads corresponding coordinates from SAL.csv or AIL.csv (cf. Table 4) and optionally visualizes them.

### Examples of the audio signals

In this section, we take a glimpse into the database by observing some of the signals in both the SAL and the AIL, which will also make evident the different acoustics of the spaces.

Figure 9 displays the waveform (top of each sub-figure) and corresponding spectrogram (bottom of each sub-figure) for a number of signals related to the SAL. The colourmap in the spectrograms corresponds to the squared magnitude of the short-time Fourier transform coefficients and is plotted in dB. Figure 9a is the first 10 s of the source signal corresponding to a female speaker, F1 (cf. Table 3). Figure 9b is a computed RIR in the SAL from the loudspeaker S0_1 to microphone BTELF (cf. Fig. 3), where the reverberation time is seen to be quite long and highly frequency-dependent. Figure 9c shows the recorded signal of the source signal F1 (from Fig. 9a) in the microphone BTELF after being played through the loudspeaker S0_1. The effect of the reverberation is evident as the spectrogram shows how the source signal has now been distorted in both time and frequency. Figure 9d is the result of a convolution between the RIR from loudspeaker S0_1 to microphone BTELF (Fig. 9b) and the F1 source signal (Fig. 9a). This signal is representative of how the recorded signal from Fig. 9c would typically be simulated. As should be expected, Fig. 9 c and d appear quite similar. However, Fig. 9e illustrates the difference (error) between the waveform plots in Fig. 9c and Fig. 9d, with the corresponding spectrogram of this error, demonstrating that the simulated signal and recorded signal are not identical. The error may be due to a variety of reasons such as acoustic noise, loudspeaker non-linearities, recording hardware limitations including slow phase drifts, cf. Section 5.2, and slowly time-variant as well as not perfectly linear sound propagation.

Figure 10 displays signals from the AIL in a similar manner to that of Fig. 9. The first 10 s of the same source signal, F1 (cf. Table 3) is observed (Fig. 10a). Figure 10b is a computed RIR in the AIL from the loudspeaker SL5_1 to microphone BTELF (cf. Fig. 3), where it can be observed that the reverberation time is significantly shorter as compared to the SAL and more uniform across frequency. Figure 10c shows the recorded signal of the source signal F1 (from Fig. 10a) in the microphone BTELF after being played through the loudspeaker SL5_1. Figure 10d is the result of a convolution between the RIR from loudspeaker SL5_1 to microphone BTELF (Fig. 10b) and the F1 source signal (Fig. 10a). Figure 10e is the difference (error) between the waveform plots in Fig. 10c and d. It can once again be observed that although the simulated and recorded signals are quite similar, they are not identical.

Figure 11 depicts the waveform and corresponding spectrogram from a 15 s sample of the cocktail party noise. The left of Fig. 11 is the signal CP2 (cf. Table 3) for microphone XM2 in the SAL and the right of Fig. 11 is the signal CP5 from XM2 in the AIL. The non-stationary behavior of this type of noise over time and frequency is quite evident.

### Reverberation times

The reverberation time $\mathrm {T_{20}}$ for the two rooms SAL and AIL is estimated at full bandwidth as well as in different octave bands. The estimate is obtained from the slope of a line fitted on the decay curves of the RIRs according to the ISO standard [41] and using the code in [42]. Here, the line was fitted in the dynamic range between − 5 dB and − 25 dB of the decay curve. A plot of the estimated reverberation times is shown in Fig. 12. As can be seen, the full-band reverberation time is significantly higher in the SAL with 2.1 s as compared to the AIL with 0.5 s. We further note that ${\mathrm T}_{20}$ in the SAL is largest between 1 and 2 kHz and quickly reduces above, while it is less dependent on frequency in the AIL. While in the AIL, the variance of the $\mathrm {T_{20}}$ estimates continuously decreases with frequency, we observe that it increases again above to 2 kHz in the SAL. This may be due to an observed magnitude decay of the SAL RIRs above 2 kHz, resulting in less accurate line fitting. In addition, the increased directivity of the loudspeakers at higher frequencies may result in stronger variations of the generated sound field with regards to the loudspeaker placement.

## Conclusion

In this paper, a database of acoustic recordings, referred to as the Multi-arraY Room Acoustic Database (MYRiAD), has been presented, which facilitates the recreation of noisy and reverberant microphone signals for the purpose of evaluating audio signal processing algorithms. Recordings were made in two different rooms, the SONORA audio laboratory (SAL) and the Alamire Interactive Laboratory (AIL), with significantly different reverberation times of 2.1 s and 0.5 s, respectively. In the SAL, a microphone configuration, M1, was used, which consists of in-ear dummy head microphones, microphones on behind-the-ear pieces placed on the dummy head, and external microphones (i.e., other microphones in the room). In the AIL, recordings were made in two different positions within the room using the microphone configuration M1 along with a second microphone configuration, M2, which consists of two concentric circular microphone arrays. In the SAL, 10 movable loudspeakers were used for sound generation, while in the AIL, a built-in array of 24 loudspeakers was used. The database contains room impulse responses, speech, music, and stationary noise signals, as well as recordings of a live cocktail party held in each room. MATLAB and Python scripts are included for accessing audio data and coordinates. The database is publicly available at [43].

## Data Availability

The database is publicly available at [43].

## Abbreviations

MYRiAD: Multi-ArraY Room Acoustic Database
RIR: Room impulse response
DH: Dummy head
BTE: Behind-the-ear
XM: External microphone
CMA: Circular microphone array
SAL: SONORA Audio Laboratory
AIL: Alamire Interactive Laboratory
CSTR: Centre for Speech Technology Research
VCTK: Voice Cloning Toolkit
EBU SQAM: European Broadcast Union Sound Quality Assessment Material

## Footnotes

1 BTE pieces are commonly used for hearing aids or cochlear implant devices. There is no additional processing done on the microphone signals before arriving to the data acquisition system.

2 A typical use-case of such XMs consists in providing additional information to improve the enhancement of BTE signals  [30–32].

3 Among others, use-cases of CMAs include signal enhancement [33] and localization [34], for instance in smart speakers, and sound zoning [13, 14].

4 Note that XM1 is taped on a stand of 18 mm diameter (holding the DH), which may impact the effective directivity pattern of the microphone at high frequencies.

5 This implies acoustic scattering effects that may not match the envisioned application. For instance, when simulations are performed using the CMAs, scattering from the DH may not be meaningful to the simulated scenario. Nevertheless, given that an accurate reproduction of scattering is hardly ever practical, this does not compromise the use of these signals to evaluate acoustic signal processing algorithms.

6 It should be noted that the estimated impulse responses also include some characteristics of the recording hardware. Consequently these impulse responses are, in a strict sense, not the true RIRs which represent the characteristics of the room only. Nevertheless these impulse responses are designated as RIRs for simplicity.

7 Only a minority of the recorded signals required a shift of at most 2 samples.

## References

[1] Loizou PC (2007). Speech Enhancement: Theory and Practice.

[2] S. Gannot, I. Cohen, Adaptive beamforming and postfiltering. Springer Handbook of Speech Processing (Springer, New York City, 2007), pp. 945–978

[3] S. Doclo, S. Gannot, M. Moonen, A. Spriet, Acoustic beamforming for hearing aid applications. in *Handbook on Array Processing and Sensor Networks* (Wiley, Hoboken, 2010), pp. 269–302

[4] Naylor PA, Gaubitch ND (2010). Speech Dereverberation.

[5] Brandstein M, Ward D (2013). Microphone Arrays: Signal Processing Techniques and Applications.

[6] Kinoshita K, Delcroix M, Gannot S, Habets EAP, Haeb-Umbach R, Kellermann W, Leutnant V, Maas R, Nakatani T, Raj B, Sehr A, Yoshioka T (2016). A summary of the REVERB challenge: state-of-the-art and remaining challenges in reverberant speech processing research. EURASIP J. Adv. Signal Process..

[7] Gannot S, Vincent E, Markovich-Golan S, Ozerov A (2017). A consolidated perspective on multimicrophone speech enhancement and source separation. IEEE/ACM Trans. Audio Speech Lang. Process..

[8] Vincent E, Virtanen T, Gannot S (2018). Audio Source Separation and Speech Enhancement.

[9] Eaton J, Gaubitch ND, Moore AH, Naylor PA (2016). Estimation of room acoustic parameters: The ACE challenge. IEEE/ACM Trans. Audio Speech Lang. Process..

[10] K. Sridhar, R. Cutler, A. Saabas, T. Parnamaa, M. Loide, H. Gamper, S. Braun, R. Aichner, S. Srinivasan, ICASSP 2021 acoustic echo cancellation challenge: datasets, testing framework, and results. in *Proc. 2021 IEEE Int. Conf. Acoust. Speech Signal Process* (Toronto, Ontario, 2021), pp. 151–155

[11] van Waterschoot T, Moonen M (2010). Fifty years of acoustic feedback control: State of the art and future challenges. Proc. IEEE.

[12] C. Evers, H.W. Löllmann, H. Mellmann, A. Schmidt, H. Barfuss, P.A. Naylor, W. Kellermann, The LOCATA challenge: acoustic source localization and tracking. IEEE/ACM Trans. Audio, Speech, Lang. Process. **28**,1620–1643 (2020)

[13] P. Coleman, P.J.B. Jackson, M. Olik, M. Møller, M. Olsen, J. Abildgaard Pedersen, Acoustic contrast, planarity and robustness of sound zone methods using a circular loudspeaker array. J. Acoust. Soc. Am. **135**(4),1929–1940 (2014) 10.1121/1.4866442 25234991

[14] Betlehem T, Zhang W, Poletti MA, Abhayapala TD (2015). Personal sound zones: Delivering interface-free audio to multiple listeners. IEEE Signal Process. Mag..

[15] J. Barker, S. Watanabe, E. Vincent, J. Trmal, The fifth ‘CHiME’ speech separation and recognition challenge: dataset, task and baselines. in *Proc. Interspeech 2018* (Hyderabad, India, 2018), pp. 1561–1565

[16] J.Y.C. Wen, N.D. Gaubitch, E.A.P. Habets, T. Myatt, P.A. Naylor, Evaluation of speech dereverberation algorithms using the MARDY database. in *Proc. 2006 Intl. Workshop Acoust. Echo Noise Control* (Paris, 2006)

[17] M. Jeub, M. Schafer, P. Vary, A binaural room impulse response database for the evaluation of dereverberation algorithms. in *Proc. 2009 16th Int. Conf. Digital Signal Process* (Santorini, 2009), pp. 1–5

[18] H. Kayser, S.D. Ewert, J. Anemüller, T. Rohdenburg, V. Hohmann, B. Kollmeier, Database of multichannel in-ear and behind-the-ear head-related and binaural room impulse responses. EURASIP J. Adv. Signal Process. **2009**, (2009), pp. 1–10

[19] R. Stewart, M. Sandler, Database of omnidirectional and B-format room impulse responses. in *Proc. 2010 IEEE Int. Conf. Acoust., Speech, Signal Process.* (Dallas, 2010), pp. 165–168

[20] J.K. Nielsen, J.R. Jensen, S.H. Jensen, M.G. Christensen, The single- and multichannel audio recordings database (SMARD). in *Proc. 2014 Int. Workshop Acoustic Signal Enhancement* (Antibes, 2014), pp. 40–44

[21] E. Hadad, F. Heese, P. Vary, S. Gannot, Multichannel audio database in various acoustic environments. in *Proc. 2014 Int. Workshop Acoustic Signal Enhancement* (Antibes, 2014), pp. 313–317

[22] W.S. Woods, E. Hadad, I. Merks, B. Xu, S. Gannot, T. Zhang, A real-world recording database for ad hoc microphone arrays. in *Proc. 2015 IEEE Workshop Appl. Signal Process. Audio, Acoust.* (New Paltz, 2015), pp. 2–6

[23] Szöke I, Skácel M, Mošner L, Paliesek J, Černockỳ J (2019). Building and evaluation of a real room impulse response dataset. IEEE J. Selected Topics Signal Process..

[24] Di Carlo D, Tandeitnik P, Foy C, Bertin N, Deleforge A, Gannot S (2021). dEchorate: a calibrated room impulse response dataset for echo-aware signal processing. EURASIP J. Audio Speech Music Process..

[25] J. Čmejla, T. Kounovskỳ, S. Gannot, Z. Koldovskỳ, P. Tandeitnik, MIRaGe: multichannel database of room impulse responses measured on high-resolution cube-shaped grid. in *2020 28th European Signal Process. Conf.* (Amsterdam, 2021), pp. 56–60

[26] S. Koyama, T. Nishida, K. Kimura, T. Abe, N. Ueno, J. Brunnström, MESHRIR: a dataset of room impulse responses on meshed grid points for evaluating sound field analysis and synthesis methods. in Proc. 2021 IEEE Workshop Appl. Signal Process. Audio, Acoust. (IEEE, New Paltz, 2021), pp. 1–5

[27] Zhao S, Zhu Q, Cheng E, Burnett IS (2022). A room impulse response database for multizone sound field reproduction. J. Acoust. Soc. Am..

[28] M. Van Segbroeck, A. Zaid, K. Kutsenko, C. Huerta, T. Nguyen, X. Luo, B. Hoffmeister, J. Trmal, M. Omologo, R. Maas, DiPCo - dinner party corpus (2019). arXiv preprint arXiv:1909.13447

[29] Fischer T, Caversaccio M, Wimmer W (2020). Multichannel acoustic source and image dataset for the cocktail party effect in hearing aid and implant users. Scientific Data.

[30] Farmani M, Pedersen MS, Tan Z-H, Jensen J (2017). Informed sound source localization using relative transfer functions for hearing aid applications. IEEE/ACM Trans. Audio Speech Language Process..

[31] Gößling N, Marquardt D, Doclo S (2021). Performance analysis of the extended binaural MVDR beamformer with partial noise estimation. IEEE/ACM Trans. Audio Speech Language Process..

[32] R. Ali, Multi-microphone speech enhancement: an integration of a priori and data-dependent spatial information. PhD thesis (KU Leuven, Leuven, 2020)

[33] Huang G, Benesty J, Chen J (2017). On the design of frequency-invariant beampatterns with uniform circular microphone arrays. IEEE/ACM Trans. Audio Speech Language Process..

[34] Pavlidi D, Griffin A, Puigt M, Mouchtaris A (2013). Real-time multiple sound source localization and counting using a circular microphone array. IEEE Trans. Audio Speech Language Process..

[35] T. van Waterschoot, KU Leuven ESAT-STADIUS Audio Research Labs (2022). https://lirias.kuleuven.be/3940173

[36] M. Holters, T. Corbach, U. Zölzer, Impulse response measurement techniques and their applicability in the real world. in *Proc. 2009 12th Int. Conf. Digital Audio Effects* (Como, 2009), pp. 108–112

[37] C. Veaux, J. Yamagishi, K. MacDonald, CSTR VCTK Corpus: English Multi-speaker Corpus for CSTR Voice Cloning Toolkit (2016). http://homepages.inf.ed.ac.uk/jyamagis/page3/page58/page58.html

[38] Anti-Everything, Federation Day. Children of a Globalised World (Musical Album) (2011). ISRC: TTA101100005.

[39] European Broadcasting Union, Sound quality assessment material recordings for subjective tests (2008). https://tech.ebu.ch/publications/sqamcd

[40] Dokmanić I, Parhizkar R, Ranieri J, Vetterli M (2015). Euclidean distance matrices: Essential theory, algorithms and applications. IEEE Signal Process. Mag..

[41] ISO 3382-1:2009, Acoustics - Measurement of Room Acoustic Parameters - Part 1: Performance Spaces. (International Organization for Standardization, Geneva, 2009), p. 26

[42] C. Hummersone, T. Prätzlich, GitHub Repository: IoSR Matlab Toolbox (2017). https://github.com/IoSR-Surrey/MatlabToolbox

[43] T. Dietzen, R. Ali, M. Taseska, T. van Waterschoot, Data repository for MYRiAD: a multi-array room acoustic database. https://zenodo.org/record/7389996 10.1186/s13636-023-00284-9 PMC10133077 37124321

